# Innovative AI methods for monitoring front-of-package information: A case study on infant foods

**DOI:** 10.1371/journal.pone.0303083

**Published:** 2024-05-16

**Authors:** Dohee Kim, Seo-Young Kim, Ra Yoo, Jaegul Choo, Hee Yang

**Affiliations:** 1 Kim Jaechul Graduate School of Artificial Intelligence, KAIST, Daejeon, Republic of Korea; 2 Advanced Institute of Convergence Technology, Suwon, Republic of Korea; 3 Department of Agricultural Biotechnology, Seoul National University, Seoul, Republic of Korea; 4 Department of Food and Nutrition, Kookmin University, Seoul, Republic of Korea; University of Agriculture Faisalabad, PAKISTAN

## Abstract

Front-of-package (FOP) is one of the most direct communication channels connecting manufacturers and consumers, as it displays crucial information such as certification, nutrition, and health. Traditional methods for obtaining information from FOPs often involved manual collection and analysis. To overcome these labor-intensive characteristics, new methods using two artificial intelligence (AI) approaches were applied for information monitoring of FOPs. In order to provide practical implementations, a case study was conducted on infant food products. First, FOP images were collected from Amazon.com. Then, from the FOP images, 1) the certification usage status of the infant food group was obtained by recognizing the certification marks using object detection. Moreover, 2) the nutrition and health-related texts written on the images were automatically extracted based on optical character recognition (OCR), and the associations between health-related texts were identified by network analysis. The model attained a 94.9% accuracy in identifying certification marks, unveiling prevalent certifications like Kosher. Frequency and network analysis revealed common nutrients and health associations, providing valuable insights into consumer perception. These methods enable fast and efficient monitoring capabilities, which can significantly benefit various food industries. Moreover, the AI-based approaches used in the study are believed to offer insights for related industries regarding the swift transformations in product information status.

## Introduction

Front-of-Package (FOP) information, which refers to the content prominently displayed on the front side of a product’s package [1], holds paramount importance by enabling consumers to easily understand products, whether in online listings or on physical store shelves [2, 3]. Based on standardized regulations, especially in the food market, FOP information becomes more standardized and refined to prevent consumers from being confused or misleading [4]. For example, the U.S. government’s Nutrition Labeling and Education Act of 1990 mandated food labeling rules, granting the Food and Drug Administration (FDA) authority to require nutrition labeling and nutrient content claims [5]. In 2013, the FDA introduced "gluten-free" certification standards [6, 7], and voluntary standards, such as organic guidelines and the Non-GMO Project Standard, also emerged.

Since product information, including FOP information, serves as a significant influencing factor in purchase decisions [8–10] and is also a crucial tool for companies to communicate their product’s value to consumers [11–13], several earlier studies sought to investigate the written information on FOPs for monitoring markets by analyzing the statement across different food categories or the alignment of statements with legal requirements, as well as scientific evidence [14, 15]. Recently, with the increasing emphasis on product quality and functional benefits, certification, nutrition, and health-related information on FOPs has also been highlighted [16, 17]. However, the manual analysis of collected data required a considerable investment of time and effort from the researchers [18], which imposed challenges in expanding the size of the data pool.

With advancements in artificial intelligence (AI)-based image data processing [18], some studies with food products have applied AI techniques for various purposes. Object detection, a part of computer vision (CV) [19], detects objects within an image and assigns labels to them, indicating what they are [20]. This technique predicts consumers’ food choices from vending machines [19] or calculates calories from food images [20]. Optical character recognition (OCR) is another type of CV technology that locates and recognizes characters from an image and converts them into machine-encoded texts [21]. This technique extracts detailed information about products, such as expiration dates [22, 23] and a list of halal ingredients for verifying accuracy [24]. However, for the purpose of monitoring the status of food products and detecting market changes, there are still few studies incorporating AI, such as object detection and OCR, to efficiently extract and analyze product information from FOP images, which may overcome the aforementioned limitations of manual methods.

Infant food products, which are one of the food categories with heightened consumer involvement, were chosen for the case study. FOPs of infant foods convey crucial nutrition and health-related information, significantly influencing purchasing decisions [25–27]. Furthermore, especially among parents who actively engage in social activities, the demand for more comprehensive and detailed information is increasing [17]. Previous studies manually analyzed infant food information, encountering limitations that arose due to the time and effort required for manual analysis [28, 29].

Here, we propose a new AI-based monitoring method for FOP information on food items in this study. This study aims to efficiently analyze the image and text information using AI techniques to comprehend the information provided on FOPs of infant food products. Applied with AI techniques of object detection and OCR, the new monitoring method has the advantage of expanding the analyzing data size and efficiently extracting different types of certification marks and various statements, including nutrition and health-related texts, from FOP images. Subsequently, we applied frequency and network analysis, as it can provide notable interpretations by quantifying and visualizing the relations between texts [30, 31]. The output from our new method is verified by analyzing the results of infant foods obtained by manual analysis in other previous studies.

## Materials and methods

### FOP data collection

FOP images of infant food products were collected from Amazon’s website (www.amazon.com). Products within the five categories of infant foods, including meals, formula, snack foods, beverages, and cereal & porridge, were collected in February 2021 using the Python web scraping library ‘Selenium’. For each product, the image, name, category, and Amazon standard identification number were crawled. During the data collection process, advertising data unrelated to infant foods were removed, as were duplicate data and product data with image quality below 50KB. The number of product image data after pre-processing was 1,176.

### Development of a certification mark detection model

An object detection model was initially developed to recognize several certification marks on FOP images using Google’s AutoML Vision to determine the certification status of infant food products. This model was selected for its ability to quickly achieve optimal performance, even with limited training data [32].

Certification marks indicate symbols highlighting a product’s characteristics that a manufacturer has verified compliance with specific standards or regulations, and the model was trained to identify four types of certification marks on FOP images: USDA Organic, Non-GMO, Kosher, and Gluten-Free. The specific criteria for each certification are presented in Table 1. USDA Organic is an organic certification mark supervised by the U.S. Department of Agriculture (USDA) and awarded to products with an organic content of over 95% [33]. Non-GMO Project Verified is a certification mark widely used in the United States for food certified by the non-profit organization, the Non-GMO Project. The certification is given to products that contain less than 0.9% genetically modified organisms (GMOs), adhering to the strictest EU standards [34]. The U-shaped Kosher certification mark, maintained by the Orthodox Union, is the most common Kosher certification mark in the United States [35]. Gluten-Free, maintained by the Gluten-Free Certification Organization (GFCO), is also widely used worldwide. GFCO only grants Gluten-Free certification if the gluten content of a food is less than 10 ppm [36].

**Table 1:** Descriptions of major food certifications used in the United States and selected for training in the study.

Certification mark	USDA Organic	Non-GMO	Kosher	Gluten-Free
**Description**	Possible for more than 95% organic contents	Food with 0.9% or fewer genetically modified organisms	Only food that complies with Jewish law	Only food containing less than 10 ppm gluten
**Competent authority**	U.S. Department of Agriculture (USDA)	Non-GMO Project	Orthodox Union	Gluten-Free Certification Organization (GFCO)

**Fig 1:**
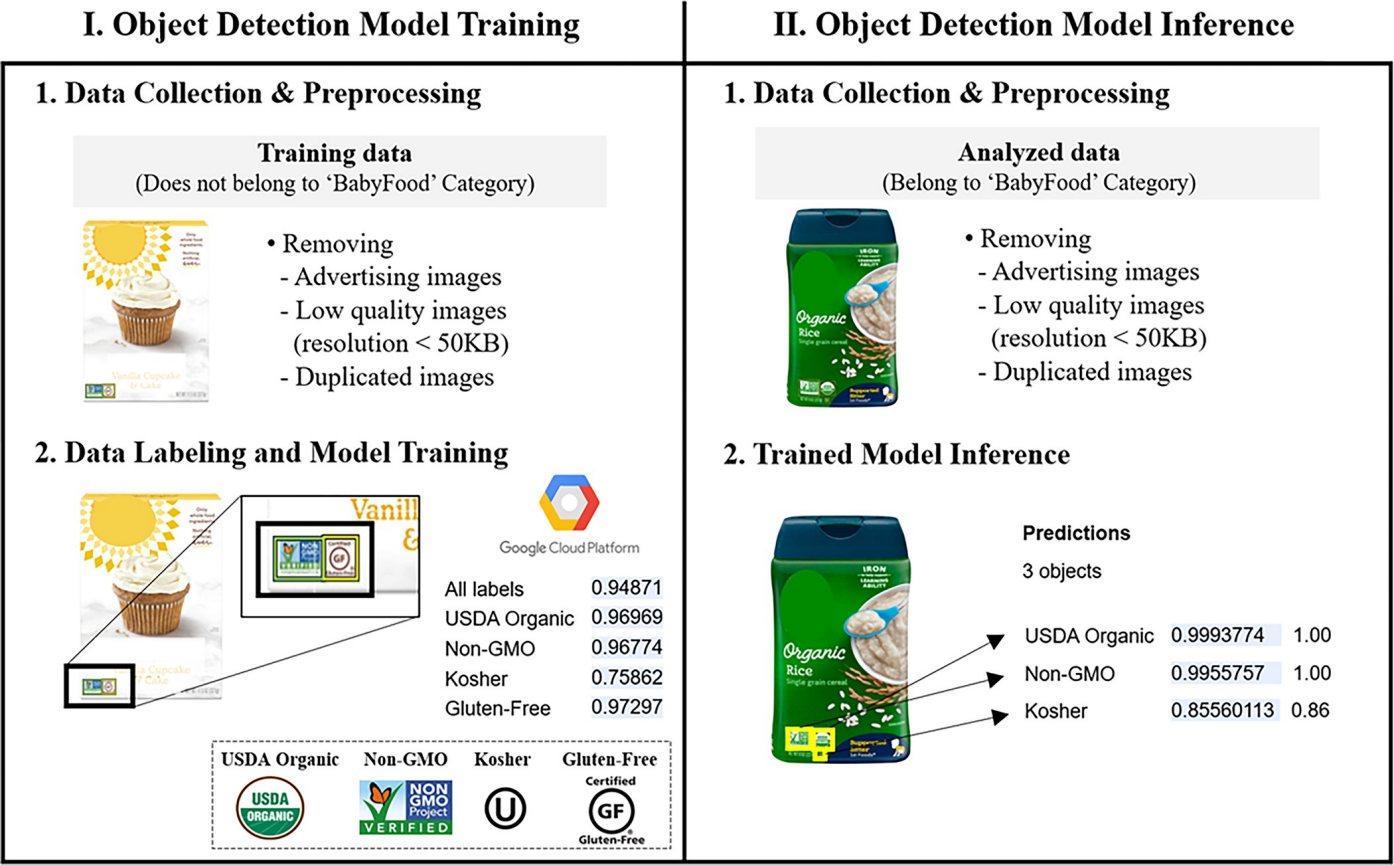
Process of an object detection model development for certification mark recognition on front-of-package images. The overall process can be divided into two distinct stages: model training and inference. During the training stage, the training dataset is collected, pre-processed, labeled with the certification marks, and used to train the model. During the inference stage, pre-processed infant food products’ images are used as the test dataset to grasp the certification status.

Fig 1 illustrates the process of training and inferencing the object detection model. The training dataset consisted of images gathered from various categories on Amazon, excluding the infant foods category. In this study, four classes were trained: USDA Organic, Non-GMO, Kosher, and Gluten-Free. Since each certification mark has a unique shape and color, as shown in Fig 1, a relatively small number of training examples were required for each class. 129 images were trained for USDA Organic, 128 for Non-GMO, 154 for Kosher, and 100 for Gluten-Free. Some of the trained images contained multiple certification marks and were thus used to train more than one class, resulting in a total of 328 FOP images used as the training dataset for the object detection model.

After uploading the entire training dataset to Google Cloud, each training image data was labeled with a bounding box indicating the location of a certification mark. The labeled data were then randomly split into three sets: 80% for training, 10% for validation, and 10% for testing.

### Data extraction and analysis

The trained object detection model was applied to the pre-processed infant food products’ FOP images. The confidence threshold—i.e., the reference probability required for the model to return a predicted value—was set at 0.5. By applying this threshold to the model’s predictions, the certification usage status of each product was determined.

**Fig 2:**
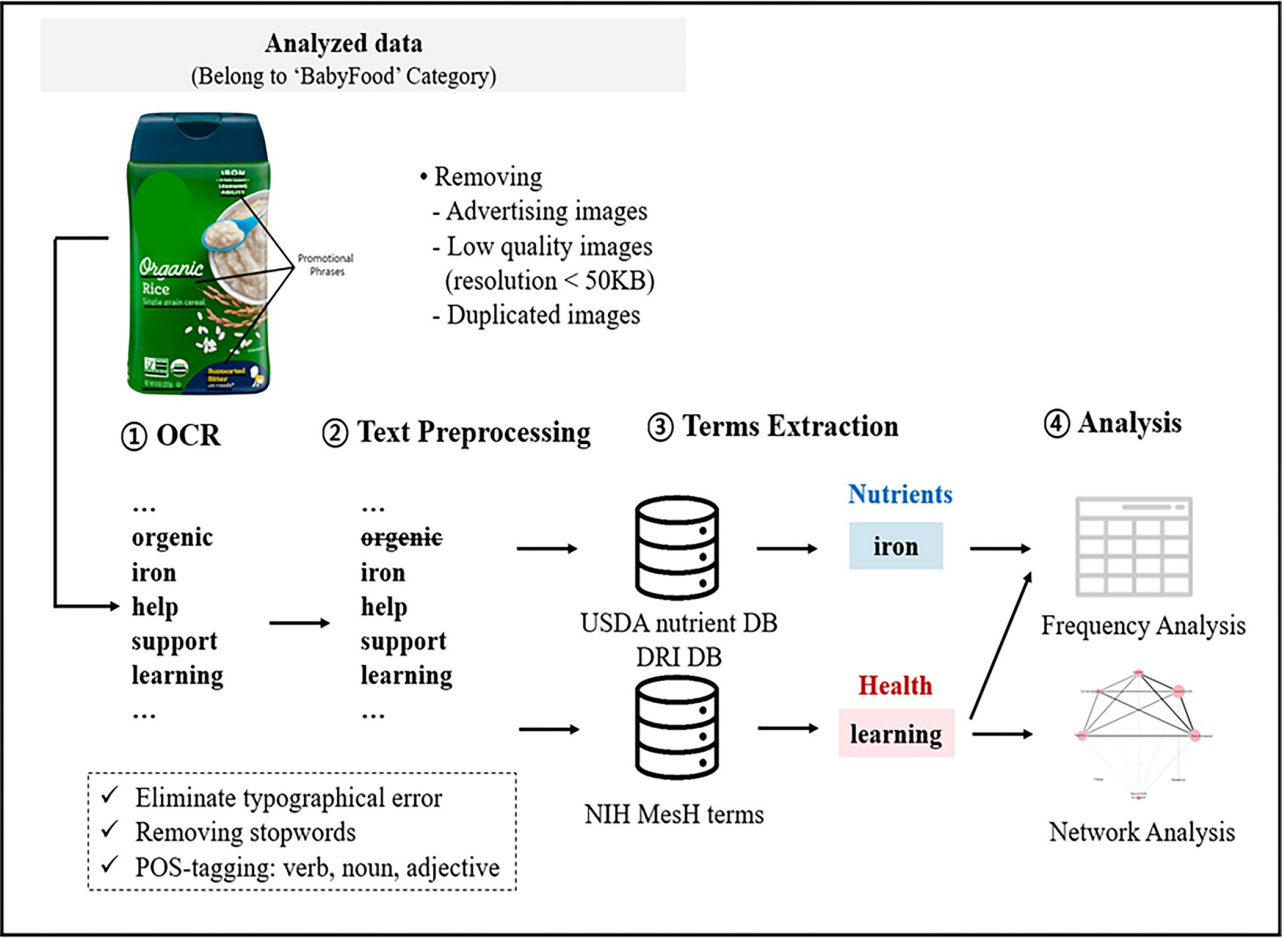
Process of nutrition and health-related text analysis for the front-of-package images using optical character recognition. From pre-processed infant food product images, the texts are extracted using OCR, then processed and filtered using nutrition and health-related databases. Following this, the nutrition and health-related texts are used for frequency analysis, and the association of co-occurring health-related texts is further identified by network analysis.

To determine the frequency of nutrition and health-related texts -the list of contained macronutrients, micronutrients, and their expected effects in terms of physical and mental health- as well as to find the relations within health-related texts of infant foods, all texts were initially extracted from the FOP image dataset pre-processed in the aforementioned FOP image data collection and pre-processing stage using Naver’s OCR application programming interface (API). This API was selected for its superior accuracy level in extracting texts [37]. Fig 2 shows the process of extracting and analyzing nutrition and health-related texts.

To confirm the accuracy of the extracted texts, the Python text pre-processing library ‘Natural Language Toolkit’ (NLTK) was used to ascertain whether the extracted words were present in the standard dictionary. Irrelevant stopwords, such as syntactic words (e.g., is, the), as well as misextracted words, were removed. Furthermore, lemmatization was performed to transform inflected words to their base forms [38]. The USDA National Nutrient Database for Standard Reference [39] and Dietary Reference Intake (DRI) [40] were used to recognize nutrition and health-related texts. Moreover, Medical Subject Headings (MeSH) terms were utilized to identify health-related texts. The extracted texts were cross-referenced with texts in the database and terminology list, and only the matching texts were included in the analysis.

Nutrition and health-related texts were classified into separate categories. Nutrition-related texts were grouped as “Macronutrients” (*carbohydrate*, *protein*, *fat*), “Vitamins” (e.g., *vitamin C*, *vitamin D*, *biotin*), “Minerals” (e.g., *iron*, *zinc*, *calcium*), “Lipids” (e.g., *DHA*, *EPA*, *cholesterol*, *choline*), “Disaccharides & Polysaccharides” (e.g., *sugar*, *fiber*) and other nutrients (e.g., *water*, *lutein*, *caffeine*). Although Lipids, Disaccharides & Polysaccharides are generally recognized as fats and carbohydrates, the two categories were not identified as part of the “Macronutrients” category in this work. This classification was based on the specific portrayal of these nutrients on product labels, as texts including *DHA* and *sugar* on the FOPs often indicated alternative interpretations. For the health-related texts, the categories were developed based on the International Classification of Functioning, Disability, and Health (ICF) [41]. The categorization was organized as follows: the categories encompassed “Brain Development” (e.g., *learn*, *brain*), “General Growth” (e.g., *grow*), “Immunity” (e.g., *immune*, *allergy*), “Digestion” (e.g., *digest*, *fussiness*, *diarrhea*), “Eye Development” (e.g., eye, *vision*), “Bone & Tooth Development” (e.g., *bone*, *tooth*), “Malnutrition” (e.g., *malnutrition*), and “Calming” (e.g., *sleep*, *anger*, *stress*).

In the first step of the analysis process, the frequency of texts belonging to each nutrition or health category was examined. Then, network analysis, which identifies meaningful patterns within complex data by analyzing the properties and structures of the network, which is a graph consisting of nodes and edges representing co-occurrences between nodes, was performed between the eight health-related categories using the ‘networkx’ library based on the co-occurrence of health-related texts.

## Results

### Certification mark usage status of infant food products

After training the certification mark detection model, an overall performance of 94.9% was obtained. Performance refers to a score that indicates the confidence level of the model for each class provided by Google. Table 2 exhibits the performances of USDA Organic, Non-GMO, Kosher, and Gluten-Free; 97%, 96.8%, 75.9%, and 97.3%, respectively (Table 2).

**Table 2:** Performance of the certification mark recognition model based on object detection developed using Google’s AutoML Vision.

Certification mark	USDA Organic	Non-GMO	Kosher	Gluten-Free	Total
**Performance**	97.0%	96.8%	75.9%	97.3%	94.9%

Table 3 presents the certification usage status of infant food products across five categories: meals, formula, snack foods, beverages, and cereal & porridge. The proportions of organic certifications in the meals category were higher than in other categories, with USDA Organic and Non-GMO accounting for 72.4% and 61.3% of products, respectively. A significant proportion of products had both USDA Organic and Non-GMO certifications. The snack foods category also showed a high proportion of USDA Organic certification, accounting for 52.3% of products. By contrast, within the beverages, cereals & porridge, and formula categories, the proportion of USDA Organic certification was around 25%. The ratio of Kosher certification varied across all categories, ranging from 49.3% in the meals category to 92.8% in the formula category. Interestingly, Non-GMO certification was not used at all in the formula category, while Gluten-Free certification was rare across all the infant product categories.

**Table 3:** Status of using certifications for infant food products.

		Infant food product category					
		Meals	Snack Foods	Beverages	Cereal & Porridge	Formula	
**Certification mark type**	**USDA Organic**	398 (72.4%)	102 (52.3%)	42 (21.5%)	35 (25.2%)	26 (26.8%)	
	**Non-GMO**	337 (61.3%)	38 (19.5%)	24 (12.3%)	35 (25.2%)	0 (0.0%)	
	**Kosher**	271 (49.3%)	136 (69.7%)	169 (86.7%)	121 (87.1%)	90 (92.8%)	
	**Gluten-Free**	4 (0.7%)	3 (1.5%)	1 (0.5%)	1 (0.7%)	0 (0.0%)	
**Total**		550 (100%)	195 (100%)	195 (100%)	139 (100%)	97 (100%)	1,176

Duplicated values were reflected for the front-of-package images containing multiple certification marks.

### Nutrition and health-related information of the infant food products

The findings of frequency analysis conducted on nutrition-related texts (Table 4) presented “Minerals” (33.3%) as the largest category, with *iron* being the most prevalent text (294), followed by *mineral* (92), and *zinc* (72). Regarding “Macronutrients” (18.1%), the analysis revealed that *protein* was the most frequently observed macronutrient (238), followed by *fat* (44) and *carbohydrate* (8). Among “Vitamins” (14.6%), while the term *vitamin* was highly frequent (215), specific types of vitamins, such as *vitamin C*, *D*, and *E*, were scarce. Within the “Lipids” (13.3%) category, the frequency was high in the order of *DHA* (173), *choline* (32), *EPA* (5), and *cholesterol* (3). The “Disaccharide & Polysaccharide” (12.1%) category consisted of two nutrients: *fiber* (103) and *sugar* (91). Lastly, the category of “Other Nutrients” (8.7%) was identified with *lutein* (44) and *caffeine* (3).

**Table 4:** Frequencies of nutrient-related texts used for the front of packages identified by optical character recognition-based text analysis.

Category (n, %)	Text	Frequency
Minerals (535, 33.3%)	iron	294
	mineral	92
	zinc	72
	calcium	42
	sodium	21
	potassium	7
	selenium	5
	fluoride	1
	iodine	1
Macronutrients (290, 18.1%)	protein	238
	fat	44
	carbohydrate	8
Vitamins (235, 14.6%)	vitamin	215
	folic acid	5
	vitamin D	5
	vitamin C	4
	vitamin E	3
	biotin	1
	niacin	1
	thiamin	1
Lipids (213, 13.3%)	DHA	173
	choline	32
	EPA	5
	cholesterol	3
Disaccharide & Polysaccharide (194, 12.1%)	fiber	103
	sugar	91
Other Nutrients (139, 8.7%)	water	85
	lutein	44
	energy	7
	caffeine	3

In terms of health-related texts (Table 5), the analysis indicated that the texts related to “Brain Development” were most frequent with a total of 328 (30.9%), followed by the texts associated with “General Growth” (323, 30.4%), “Immunity” (157, 14.8%), and “Digestion” (146, 13.7%). The texts related to “Eye Development” (73, 6.9%) occurred with relatively lower frequency, and those related to “Bone & tooth development” (23, 2.2%), “Malnutrition” (10, 0.9%), and “Calming” (3, 0.3%) were also observed.

**Table 5:** Frequencies of health-related texts used for the front of packages identified by optical character recognition-based text analysis.

Category (n, %)	Text	Frequency
Brain Development (328, 30.9%)	learn	173
	brain	155
General Growth (323, 30.4%)	grow	323
Immunity (157, 14.8%)	immune	95
	allergy	62
Digestion (146, 13.7%)	digest	88
	fussiness	22
	diarrhea	15
	tummy	14
	gastrointestinal	6
	stomach	1
Eye Development (73, 6.9%)	eye	70
	vision	3
Bone &Tooth Development (23, 2.2%)	bone	19
	tooth	4
Malnutrition (10, 0.9%)	malnutrition	10
Calming (3, 0.3%)	sleep	1
	anger	1
	stress	1

**Fig 3:**
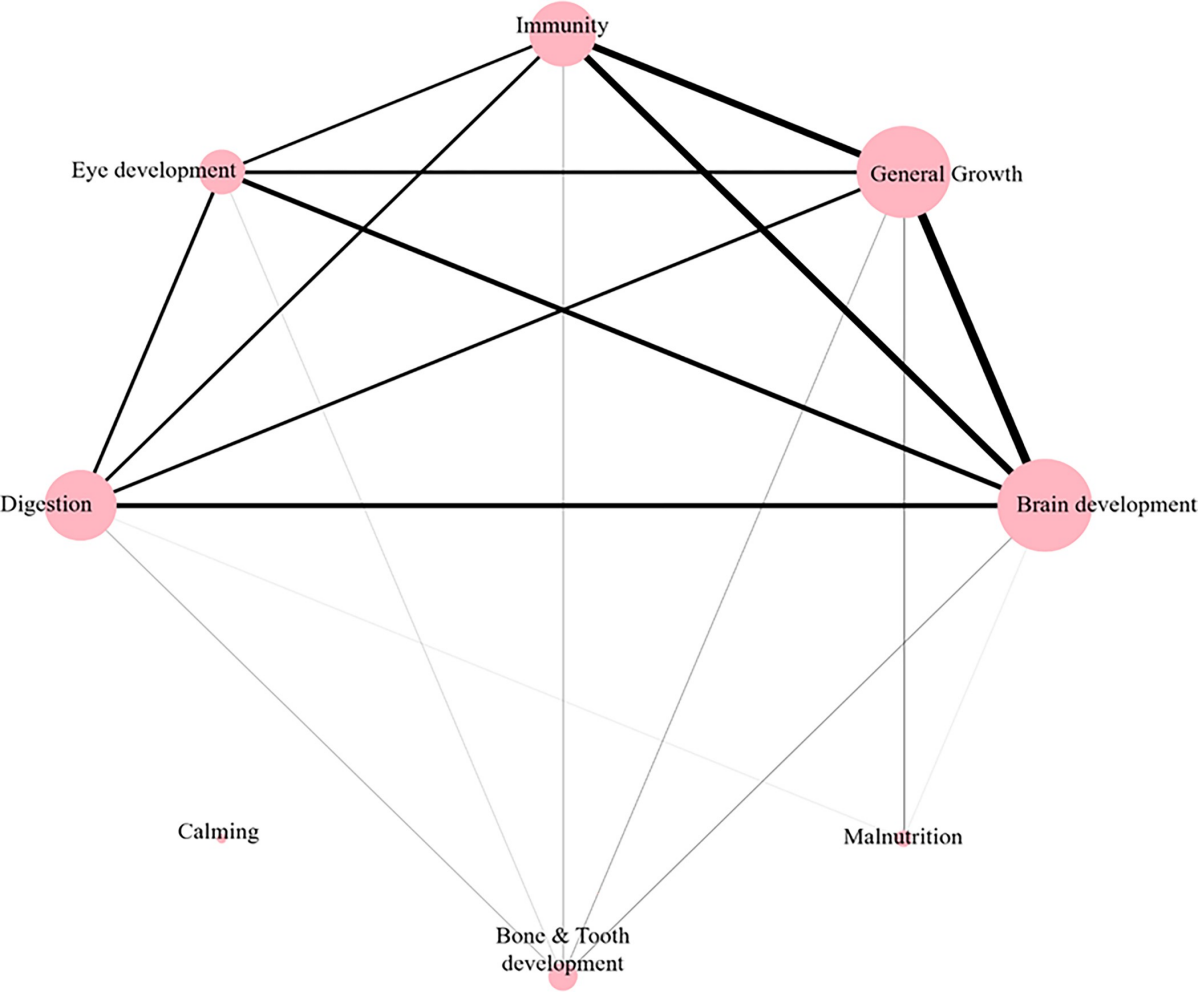
Co-occurrence network of health-related categories for baby food products.

Fig 3 describes the relationship between health-related texts on infant food products identified by their co-occurrence frequencies. Strong correlations were found in the occurrences of specific health-related texts such as “Immunity”, “General Growth”, and “Brain Development”, as indicated by the thickness of the lines in the network. Also, the occurrence of “Eye Development” and “Digestion” correlated with those of other health-related texts was observed, including “Brain Development” and “General Growth”. However, the “Calming” category consisted of the most uncommon texts and had no connections with other categories.

## Discussion

This study introduces innovative AI techniques for efficient monitoring of FOP information, overcoming limitations associated with traditional manual collection and analysis methods. By conducting a case study on infant food products, the research utilizes object detection to identify certification usage. Sequentially, optical character recognition (OCR) is applied to extract nutrition and health-related texts, while frequency or network analysis provides the frequencies or associations among this information. These approaches for identifying product information statuses are expected to offer related industries with insights into the rapidly transforming market.

The utilization of two methods—one for analyzing certification marks and another for nutrition or health-related texts—applied to assess infant food products on the American Amazon platform revealed a trend consistent with previously studied outcomes through conventional research methods. This alignment underscores the validation of both models’ efficacy and sufficient accuracy in this analysis. As presented in Table 3, the object detection model provided a comprehensive certification usage status, demonstrated with a case study of infant food products. The study found that at least half of the analyzed infant foods were certified as organic, either through USDA Organic or Non-GMO, which was consistent with the results of Cheikhrouhou et al.’s study [42]. Notably, it was also confirmed that a substantial number of products in the meals category had both USDA Organic and Non-GMO certifications, supporting the findings of Castellari et al. [34]. Also, we confirmed the efficacy of OCR-based text mining in the analysis of the information on FOPs. By conducting a frequency analysis of nutrition and health-related texts (Tables 4 and 5), the most commonly occurring phrases were identified. The findings indicated that the texts *iron* (in “Minerals”), *protein* (in “Macronutrients”), *vitamin* (in “Vitamins”), and *DHA* (in “Lipids”) were frequently mentioned in the FOPs of infant and toddler food products, which aligned with previous studies emphasizing the importance of these nutrients for early childhood development [43–45]. Especially, the observation of *iron* and *protein* as the most frequently occurring nutrients in the FOPs of infant foods (Table 4) supported the benefits of these nutrients for the development of infants and toddlers [46–48], as well as suggested supplementation of iron and protein for breast milk [49]. The study also exhibited consistent results with previous work by Berry & Gribble [50], which analyzed health information from online advertisements of infant formula, and the main claims included supporting brain and eye development, bone growth, and immune system, as well as aiding digestion.

Intriguingly, besides the statement based on legal or scientific evidence, the FOP information also partially reflects general consumers’ perceptions. The presence of the Kosher certification mark was found to be widespread in all categories of infant food products. This finding supports previous research conducted by Della Corte et al. [51], emphasizing North America’s substantial contribution to the global Kosher product market. Cohen et al. [52] and Lytton [53] suggested that Kosher certification serves as an indicator of product safety and quality to consumers—thereby impacting their buying choices—regardless of their religious convictions. The data analysis conducted in this study further strengthens the notion that Kosher certification operates as a widely recognized promotional indicator for infant food products compared to other certifications. Moreover, the co-occurrence network analysis of each category type in Fig 3 revealed significant associations among the health-related texts. Notably, “Brain Development” frequently appeared alongside “Immune” or “General Growth” categories. This association could be linked to parental perceptions associating these claims with healthier infant foods [54, 55]. Addressing parental concerns, FOPs often contain information catering to these expectations. Belamarich et al. [56] noted a prevalent advertisement trend regarding digestion-related claims in US infant formula products, consistent with this study’s findings. Likewise, our method, the visualization of the co-occurrence network between text information of FOPs, could offer intuitive comprehension, as a starting point for new research endeavors. This underscores the need for further investigation to understand manufacturers’ practices on FOPs better, particularly considering consumers’ perceptions in the development of infant foods.

Our proposed approach reduces time and manual efforts over traditional monitoring methods across the entire process, from data collection and pre-processing to analysis. Previously, it took several weeks to months to collect FOPs by purchasing or photographing a substantial number of food products, ranging from over 300 [57] to as many as 3,000 [58], in offline stores. On the other hand, we crawled 1,176 infant products on Amazon within a few hours. Also, in contrast to previous studies, which restricted the scope of collected products due to manpower limitations, this study thoroughly examined all products within the infant product category. In the pre-processing phase after data collection, previous studies manually filtered out duplicate products by capturing images of shopping carts, providing a quick overview of the collected products. In contrast, we automatically removed them using the products’ unique Amazon standard identification numbers. Additionally, instead of manual data entry for information on FOPs, the implementation of OCR enhanced efficiency, reducing human typing errors. It allowed for rapid processing of vast data volumes, optimizing resources for comprehensive market understanding. Furthermore, the certification status of infant foods was analyzed easily using an object detection model. The development of this model, based on Google’s Auto ML, required a small amount of training dataset, consisting of 328 FOP images. Although it involved labeling a total of 511 instances of four certification marks, taking several hours initially, once the model was developed, it became available for continuous use, even when dealing with large volumes of data. Therefore, it enabled quick model training and application, requiring minimal manual effort.

However, there remains careful consideration for data collection when we utilize such an AI-based monitoring method as our model because data availability may limit the method’s scope; focusing on high-market-share brands could affect the result of the analysis. Nevertheless, the utilization of AI-based monitoring methods will become more feasible in the future. The evolving consumer perception of food quality is expanding to encompass functional, social, cultural, and ethical considerations, resulting in diverse certification marks [34]. Additionally, FOPs increasingly incorporate various information such as additives and allergens, even introducing the concept of ‘advice’ through color in Nutrient Facts, which are previously listed on the back of the package as a text, demonstrating a broader range of details. This may help food companies monitor what information competitors convey through FOPs especially when exporting food. Additionally, although governmental efforts standardized mandatory and voluntary labeling, the online shopping era introduced varied information beyond FOPs, revealing issues like insufficiently presenting essential data or exaggerating health claims restricted on FOPs [28, 59]. Regulatory authorities can leverage these methods to acquire extensive fundamental data on a large scale, serving as a crucial foundation for the development of well-informed policies and regulations. Consequently, it is suggested that our AI-based monitoring methods developed in this study can be a basis model to facilitate further research in these directions.

## Conclusions

This research introduced innovative AI-based methods for product investigations, employing object detection and OCR techniques, and exemplified through a case study on infant food products. The object detection model achieved an accuracy performance rate of 94.9% in identifying certification marks, revealing prevalent certifications like Kosher across various infant food categories. Furthermore, the frequency and network analysis of nutrition and health-related texts shed light on common nutrients such as iron and protein, and health associations among brain development, immune, and general growth found on FOP labels in infant food products. These findings highlighted the benefits of employing these AI techniques to alleviate the labor-intensive challenges in prior studies. Additionally, given their capacity for comprehensive market analysis, these techniques suggest promise for further research, encompassing the monitoring of various types of information across wide range of product categories.

## Supporting information

S1 File Detailed listings of data sources and specific AI platforms with corresponding URLs for our method. (DOCX)
