# Supplementary material for: Innovative AI methods for monitoring front-of-package information: A case study on infant foods
Source: PLoS One. 2024 May 16;19(5):e0303083. doi: 10.1371/journal.pone.0303083 (PMC11098498; doi:10.1371/journal.pone.0303083)
Supplement: S1 File — (DOCX) [file pone.0303083.s001.docx]

Detailed Information on Data Sources and Methodology

▣ Materials and Methods > FOP data collection

- Meals : Amazon.com > Department > Baby Products > Feeding > Baby Foods > Meals

- Formula : Amazon.com > Department > Baby Products > Feeding > Baby Foods > Formula

- Snack Foods : Amazon.com > Department > Baby Products > Feeding > Baby Foods > Snack Foods

- Beverages : Amazon.com > Department > Baby Products > Feeding > Baby Foods > Beverages

- Cereal & Porridge : Amazon.com > Department > Baby Products > Feeding > Baby Foods > Cereal & Porridge

▣ Materials and Methods > Development of a certification mark detection model

- Google’s AutoML Vision : <https://cloud.google.com/vision/automl/docs>

- Or you can use new tool of Google’s Vertex AI : https://cloud.google.com/vertex-ai/docs/training-overview

▣ Materials and Methods > Data Extraction and analysis

- Naver’s OCR : https://www.ncloud.com/product/aiService/ocr/?language=en-US
